# Iminosugars of the Invasive Arboreal *Amorpha fruticosa* and Glycosidase Inhibition Potential

**DOI:** 10.3390/plants14142205

**Published:** 2025-07-16

**Authors:** Robert J. Nash, Barbara Bartholomew, Yana B. Penkova, Ekaterina Kozuharova

**Affiliations:** 1 PhytoQuest Limited, Plas Gogerddan, Aberystwyth SY23 3EB, UK; robert.nash@phytoquest.co.uk (R.J.N.); barbara.bartholomew@phytoquest.co.uk (B.B.); y.penkova@phytoquest.co.uk (Y.B.P.); 2 Department of Pharmacognosy, Faculty of Pharmacy, Medical University of Sofia, 1000 Sofia, Bulgaria

**Keywords:** glycosidase inhibition assay, iminosugars and iminosugar acids, antihyperglycemic activities, pipecolic acids, sustainable use

## Abstract

*Amorpha fruticosa* L. (Fabaceae) originates from North America and has become an aggressive invasive plant in many parts of the world. It affects the local biodiversity in many negative ways. Our previous in vivo tests of purified extract of *A. fruticosa* pods for antihyperglycemic activity in streptozotocin-induced diabetic spontaneously hypertensive rats (SHRs) revealed that the oral administration of purified extract of *A. fruticosa* (100 mg/kg) for 35 days to SHRs caused significant decreases in the systolic pressure, blood glucose levels, and MDA quantity. The aim of this experimental study is to test the glycosidase inhibition of several extracts of *A. fruticosa* pods. Methods: GC-MS, NMR, and a glycosidase inhibition assay were performed. Results: The results demonstrate strong inhibition of yeast alpha- and almond beta-glucosidases, rat intestinal hexosaminidase, and bovine beta-glucuronidase, but not of some other glycosidases. The activity is probably due at least in part to the presence of iminosugars and iminosugar acids. We here report on further analysis and activity assessments of *A. fruticosa* pods and leaves collected in Bulgaria, and for the first time discover glycosidase inhibitors, pinitol, and hydroxylated pipecolic acids in the species and more complex iminosugar-like compounds that may all contribute to antidiabetic potential. Hydroxylated pipecolic acids are probable precursors of iminosugars and common in legumes containing them. Considerable chemical variation was observed over four pod collections. Conclusions: *A. fruticosa* pods and leaves were found to contain a number of compounds that could contribute to the potential antihyperglycemic activities including pinitol and a complex mixture of iminosugar-related compounds derived from pipecolic acids and prolines. The pods and leaves caused potent selective inhibition of glucosidases and hexosaminidases and beta-glucuronidase. The variation between the collections might reflect the sites differing or wide phenotypic versatility allowing the success of the species as an invasive plant.

## 1. Introduction

*Amorpha fruticosa* L., a member of the Fabaceae family, is a deciduous shrub. The flowers are numerous and densely clustered in upright spikes. They are single-petaled, purplish-blue with fragrant and nectareous orange anthers. The fruits are small glandular legumes (pods) that hold one or two brownish seeds. *A. fruticosa* originates from North America [1,2,3]. This plant is commonly referred to by various names such as false indigo-bush, desert false indigo, and bastard indigo-bush. *A. fruticosa* was introduced to Europe in the early 18th century as an ornamental plant and later appreciated for its role in honey production [4,5,6,7] and soil stabilization [8]. Over time, these intentional introductions have led to its uncontrolled proliferation and its designation as a highly invasive alien species globally, spreading beyond its native range and invading regions across Africa, South America, Asia, and Europe [9,10]. One of the difficulties in controlling its invasion is that beekeepers deliberately continue planting *A. fruticosa* [11,12].

Since the plant is native to North America, it is interesting to find out its traditional medicinal applications. The Seminoles used it against gastric distress and as a tonic in general, and they also mixed it with other plants to treat chronic sickness [13]. Later on, several pharmacological effects of *A. fruticosa* were reported, such as antimicrobial (antibacterial and antifungal) activity and wound healing effects; antioxidant and acetylcholinesterase inhibition properties; anti-inflammatory and anti-tumor activities; and hepatoprotective and osteoclast inhibitory effects. The antidiabetic properties of *A. fruticosa* deserve particular attention because diabetes leads to severe complications such as diabetic neuropathy, diabetic micro and macro angiopathy, diabetic nephropathy and diabetic retinopathy, cardiovascular disease, leg amputations, stroke, chronic renal failure, vision loss, and nerve damage, and last but not least has a significant mortality rate. Therefore, diabetes has become a major focus for health systems globally. Additionally metabolic syndrome, a cluster of conditions, including obesity, dyslipidemia, type 2 diabetes, and hypertension, elevates the risk of cardiovascular disease and mortality [14].

The plant contains isoflavonoids, and their derivatives called rotenoids. The second important group of phenolic compounds is prenylated stilbenoids including amorfrutins, reported by Weidner et al. [15] to be potential antidiabetic agents and possibly functioning via being PPARγ agonists with high binding affinity. The potential for *A. fruticosa* as a treatment for diabetes and metabolic disease seems promising [14]. Later on, in vivo tests were performed with plant extract obtained by exhaustive percolation with dichloromethane and further extraction with 80% methanol. The extract of *A. fruticosa* pods (EAFp) containing flavonoids (rutin) and a minimal amount of amorfrutins (A and B) seemed to reduce insulin resistance, similar to the reduction that has been observed with the clinical drug rosiglitazone [16]. Simeonova and coauthors studied the safety of administration of EAF (100 mg/kg) fed to rats for 35 days and reported decreases in the systolic pressure, blood glucose levels, and MDA quantity. It also increased the hepatic level of the endogenous antioxidant GSH, not only in streptozotocin-induced diabetic spontaneously hypertensive rats, but also in a control group [16]. The extract was nontoxic when administrated in doses less than 1500 mg/kg but mortality was 100% at 5000 mg/kg. Although amorfrutins are reported to reduce blood glucose [15,17], they seemed to be at a low concentration in the extract tested in this experiment [16], yet that extract still had hypoglycemic activity. It has previously been shown that stilbenes can be inhibitory to yeast alpha-glucosidase as well as porcine alpha-amylase and human pancreatic lipase [18].

Legume species often accumulate bioactive small nitrogen-containing molecules including non-protein amino acids and iminosugars, and the aim of this study was to extract and identify such compounds from the pods and leaves of *Amorpha fruticosa* and look for site-to-site variability. Glycosidase inhibition activity studies were conducted on the plant extracts because iminosugars can be potent inhibitors and have formed the basis of drugs for diabetes such as Glyset (Miglitol) [19].

## 2. Results

### 2.1. Major Compounds of Amorpha Fruticosa Samples

#### 2.1.1. Chromatography–Mass Spectrometry (GC-MS)

**Figure 1:**
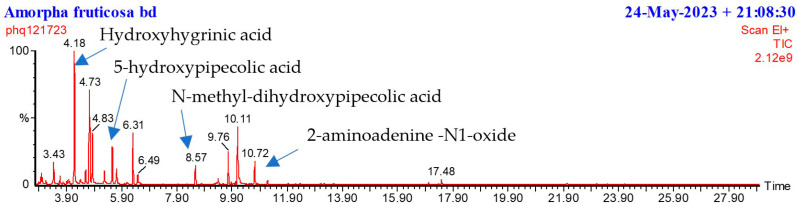
GCMS chromatogram of a pod cation exchange-retained fraction showing hydroxyhygrinic acid (4.18 min), 5-hydroxypipecolic acid (5.3 min), and an N-methyl-dihydroxypipecolic acid (8.57 min) (tms). 2-aminoadenine-N1-oxide is seen at 10.72 min.

GCMS analysis using trimethylsilylation derivatization of the cation exchange-retained fraction of *Amorpha fruticosa* pods (Figure 1) showed a few major compounds which, on further analysis, were found to be hydroxylated pipecolic acids and prolines (see Supplementary Material). Pipecolic acids are often present with iminosugars and are probably precursors in some plants [20,21].

#### 2.1.2. Nuclear Magnetic Resonance (NMR)

**Figure 2:**
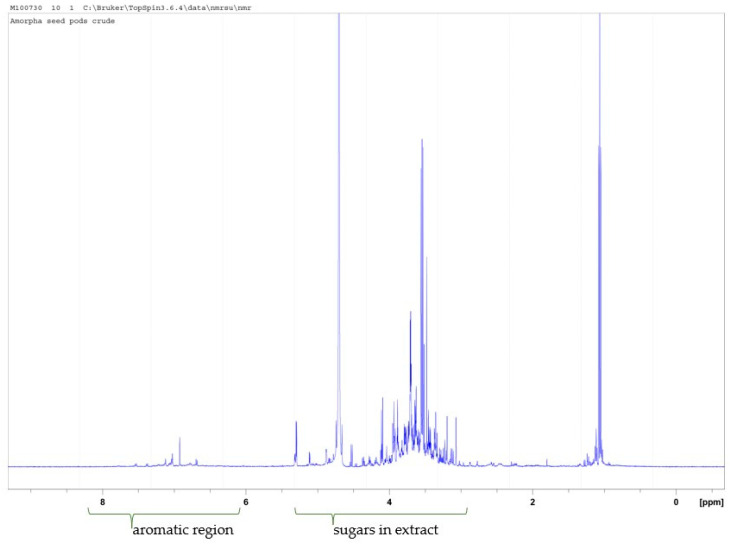
The 500 MHz proton NMR spectrum of *A. fruticosa* pod 50% aq ethanol extract run in D2O showing mainly sugars and some aromatics.

The proton NMR spectrum of *A. fruticosa* pod 50% aq ethanol extract showed relatively small amounts of aromatics (including rotenoids) at 6–8 ppm. The main components are sugars and amino acids (Figure 2).

**Figure 3:**
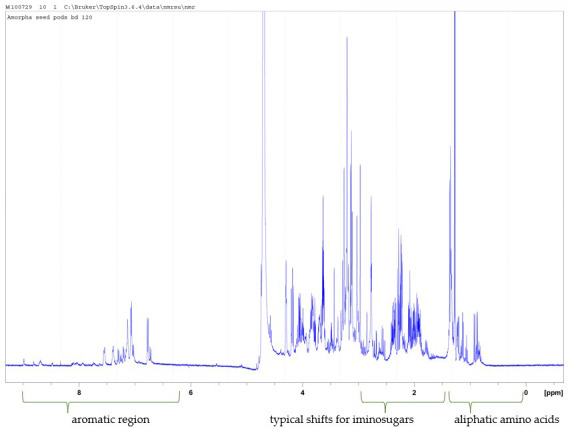
500 MHz proton NMR spectrum of the cation exchange resin-bound fraction of *A. fruticosa* run in D2O showing increased aromatics and a very complex mixture of hydroxylated small nitrogen-containing molecules.

The proton NMR spectrum of the cation exchange resin-bound fraction of *A. fruticosa* showed aromatics increased and also a complex mixture of amino and imino compounds (Figure 3).

Table 1 shows the extractable weights in methanol and 50% aq ethanol and the total amino acid (and related small nitrogen-containing compounds such as alkaloids) retained by the cation exchange resin. The extractable weights were similar but the composition of nitrogen-containing molecules showed variability as did the pinitol content. Pinitol is an osmoregulant and was particularly high in the leaves. It was determined by the distinctive tms mass spectrum with ions 147 (100%), 217 (90%), 260 amu (80%), and 443 and 449 (both 10%), with a retention time of 9.4 min and the use of a reference compound.

**Figure 4:**
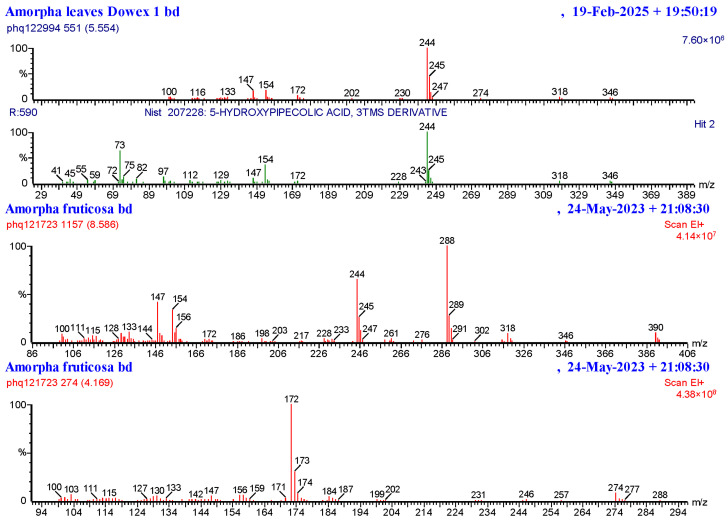
Mass spectra of hydroxylated pipecolic acids or related compounds (**top**: 5-hydroxypipecolic acid; **middle**: N-methyl-4,5-dihydroxypipecolic acid [tentative]; and **bottom**: hydroxyhygrinic acid).

These hydroxylated pipecolic acids showed variability (Figure 4). Namely, 5-hydroxypipecolic acid could be clearly seen in pod sample 1 and the leaves but not in the other pods. This compound has a distinctive trimethylsilyl-derivative (retention time 5.5 min in the conditions used) with ions 346 (10%), 318 (2%), 244 (100%), and 154 (30%) amu (145 + 3 tms to give 361 daltons with a loss of methyl (15) to 346 amu). This pipecolic acid is most probably either 4-hydroxy- or 5-hydroxy-pipecolic acid (as is common in legumes [22]), with a perfect NIST library match for the later, but the epimeric compounds give very similar spectra. A second pipecolic acid derivative was tentatively identified at 8.6 min with characteristic ions 390 (10%), 288 (100%), 244 (70%), and 154 (30%) amu. This second pipecolic acid probably had two hydroxyls and a methyl (m.wt 177 + 3 tms = 391 daltons). 4,5-dihydroxypipecolic acids are known to be found in legumes [22] and the methyl could be on the nitrogen. A further possible *N*-methyl-pipecolic acid that appeared to be an isomer of glabrin (reported as antimicrobial from *Pongamia pinnata*) was also seen [23]. The 500 MHz NMR spectroscopy of the nitrogen-containing components of the *A. fruticosa* pods showed a very complex mixture of compounds including aromatic signals but appeared to show 2-aminoadenine-N1-oxide in the mixture as a major singlet seen at 7.6 ppm with aliphatic signals obscured. 4-Hydroxyhygrinic acid was also possibly seen with a singlet of N-Me at 2.9 ppm and multiplet at 4.5 ppm.

4-Hydroxyhygrinic acid (an N-methyl-proline derivative) and 2-aminoadenine-N1-oxide were identified by using reference compounds isolated from *Casearia sylvestris* and *Trifolium pratense* respectively. An epimer of 4-hydroxyhygrinic acid with an identical tms mass spectrum but a longer retention time (4.19 instead of 3.6 min) was at a higher quantity than the epimer at 3.6 min and might be assumed to be 3-hydroxyhygrinic acid. Both compounds gave tms mass spectra with major ions 172 and 274 amu with the small molecular ion seen at 289 prior to the loss of methyl. Proline, tyrosine, and glutamic acid were identified by using authentic reference compounds and distinctive mass spectra. A few unknown iminosugar-like compounds were observed in the cation exchange-retained fractions and had ions 172 (20%) and 186 (100%) in common with proline but longer retention times (e.g., one at 9.7 min with additional fragments 302, 376, 404, and 419 amu). These nitrogen-containing compounds did not give matches in our 200 iminosugar compound spectral database or the NIST spectral database but showed distinctive features of iminosugars such as losses of 90 amu (hydroxy-tms), 102 amu (hydroxymethyl-tms), and 116 amu (carboxyl-tms). These compounds therefore appear to be novel and warrant further purification and elucidation.

### 2.2. Glycosidase Inhibition Assay

Table 2 shows the glycosidase inhibition studies conducted with the extracts and ion exchange fractions of *A. fruticosa* pods (sample 4) and leaves (sample 5). It was notable that all the pod crude methanol and 50% aq ethanol extracts gave potent inhibition of yeast alpha-glucosidase and this was shown to be dose-dependent by dilution of the pod extract. The selectivity of the inhibition was evidenced by the lack of potent inhibition of the rat intestinal alpha-glucosidase but strong inhibition of an almond beta-glucosidase. Weak inhibition of rat beta-galactosidase activities was seen with the pods; there was a slight promotion of alpha-mannosidase activity but again a notable potent inhibition of rat intestinal hexosaminidase and bovine liver beta-glucuronidase.

The cation exchange-retained fractions of both the pods and leaves also showed strong inhibition of the yeast alpha-glucosidase, suggesting nitrogen-containing molecules might be involved. Using the anion exchange resin, it appeared that alpha-glucosidase inhibition activity was unretained, suggesting basic compounds without carboxyl-groups or aromatic moieties, which tend to be retained by the strongly basic anion exchange resin. The leaves showed an inhibition of the rat intestinal alpha-glucosidase that was primarily in the anion exchange resin-unretained fractions, suggesting again basic active compounds; this activity correlated with one compound with a GC retention time of 10.17 min and distinctive ions 147 (60%), 256 (20%), 346 (50%), and 361 (20%) amu (with no NIST, or in-house, library matches); the proton NMR showed just aliphatic signals similar to 4-hydroxyhygrinic acid. The pods also showed an increased inhibition of the rat intestinal alpha-glucosidase after anion exchange chromatography. The pods’ inhibition of the hexosaminidase and beta-glucuronidase was also exclusively in the anion exchange resin-unretained fraction.

## 3. Discussion

D-Pinitol (3-O-methyl-D-chiro-inositol) was detected in the pods and leaves of *Amorpha fruticosa*. It is a cyclitol nearly ubiquitous in the Leguminosae and Pinaceae families. It plays an important role in plants as a physiological cellular modulator and chemical defense against unfavorable environmental conditions, such as water deficits and high levels of salinity. Plants rich in D-pinitol are being used in traditional medicine as empirical treatments for diabetes, inflammation, cancer, and infections [24]; the compound is widely claimed to be helpful for energy balance in athletes.

Other researchers working on *A. fruticosa* do not seem to have studied the nitrogen-containing molecules or their glycosidase inhibition [25,26].

The discovery of hydroxylated pipecolic acids in *A. fruticosa* pods and leaves is of interest because there are reports of antidiabetic activity of 4-hydroxypipecolic acid [27]. Cis-5-hydroxy-pipecolic acid is also known to occur in the antidiabetic plant *Morus alba* [28] along with iminosugars such as 1-deoxynojirimycin, which is a potent alpha-glucosidase inhibitor and the basis of the antidiabetic drugs Glyset and Miglitol. 1-DNJ and Miglitol can reduce post-prandial blood glucose levels by inhibiting a range of intestinal glucosidases [19]. While many researchers of blood sugar control just consider yeast alpha-glucosidase and compare its inhibition to that of the drug Acarbose, we here studied many more glucosidases as there are many in the human body and yeast alpha-glucosidase is in fact not strongly inhibited by Acarbose [29]. Cis-5-hydroxypipecolic was tentatively identified in *A. fruticosa* along with a possible isomer of glabrin from *Pongamia* that was reported recently to be antibacterial [23].

There are many papers on the biosynthesis of DNJ and suggestions that glucose is involved [30,31], or other precursors, but a common feature of iminosugar-containing plants is pipecolic acids. Iminosugars occur in legume genera of many of the Sophoreae along with pipecolic acids, e.g., *Baphia*, *Alexa*, *Angylocalyx*, *Castanospermum*, *Xanthocercis*, *Myroxylon*, and *Myrospermum* [32]. Harris and coauthors [20] reported that the iminosugar mannosidase inhibitor swainsonine (in legume genera *Swainsona* and *Astragalus*) was produced via lysine and pipecolic acid in *Rhizoctonia leguminicola*. Recent research has also established that the pipecolate pathway, a three-step biochemical sequence from l-lysine to *N*-hydroxypipecolic acid (NHP), is central for plant systemic acquired resistance (SAR) [33]. The iminosugar amino acid idoBR1 is a trihydroxy-pipecolic acid and although not inhibitory to glucosidases it is attracting interest as an inhibitor of sialidase and for anti-inflammatory activity with benefits for osteoarthritis and potentially brain inflammation related to neurodegeneration [34].

The analysis of the pods and leaves of *A. fruticosa* has shown potent inhibition of various glycosidases. Hydroxylated pipecolic acids and prolines occur, in addition to other components lacking carboxyls (not retained by strongly basic anion exchange resins) that give selective glycosidase inhibition. Pipecolic acids of plants have also been shown to control blood glucose in maltose challenge in rodents without being glucosidase inhibitors [35]; it would be worth trying a wider range of pipecolic acids and iminosugars in rodent studies where in vivo activity may appear that was not obvious from in vitro glucosidase assays. It seems likely that the antidiabetic activity of *A. fruticosa* is due to multiple components.

Potent and selective inhibition of alpha-glucosidases could be important for the control of blood sugar, but the potent inhibition of hexosaminidases and beta-glucuronidase is also very interesting in relation to potential applications for various disorders. Inhibition of beta-glucuronidase is associated with improved toxin excretion and the enzyme activity is elevated in diabetes, colon cancer, and periodontal disease, amongst other illnesses [36,37]. This enzyme activity is also involved in the degradation of glycosylaminoglycans that help keep skin wrinkle-free, so the inhibition may support reducing signs of aging. Inhibition of hexosaminidase activity has been reported as a new strategy for preventing or even reversing cartilage degradation in patients with osteoarthritis [38]. Hexosaminidase activity in serum increases with various illnesses and is associated with cancer cell metastasis [39]. Changes in hexosamine metabolism are also associated with cardiovascular disease [40].

## 4. Materials and Methods

### 4.1. Plant Material

**Figure 5:**
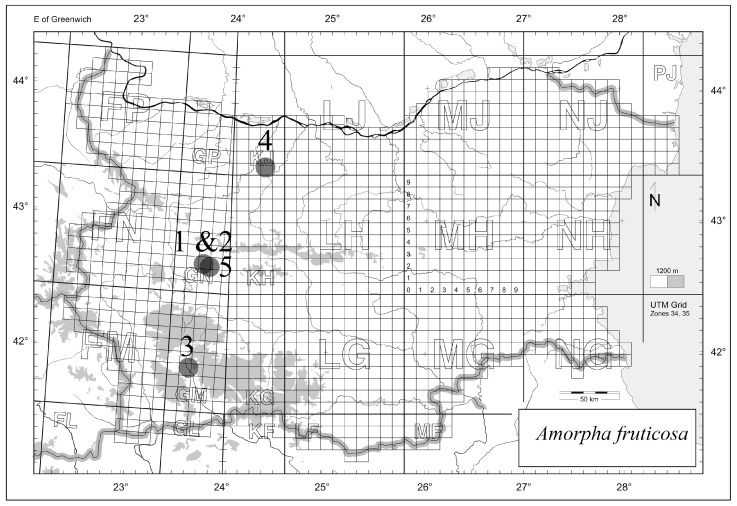
Plant sampling sites.

Pods were collected from locations 1–4 below and leaves were collected from location 5. The pods were collected from Ognjanovo village Elin Pelin District 20 November 2022, Ognjanovo village 5 October 2022, Eleshnitza village Bansko District 30 November 2022, and Gorni Dnbnk Dam Pleven District 6 January 2023; the leaves were collected from near Karapolci 3 September 2023, (Figure 5).

Dried *A. fruticosa* pods and leaves were ground and extracted for 15 h in either methanol or 50% aq. ethanol. The extracts were filtered and freeze-dried for extraction weighing, glycosidase assays, and GCMS and NMR analysis. The 50% aq. ethanol extracts were fractionated using an excess of strongly acidic cation exchange resin Amberlite IR120 in the hydrogen form with retained nitrogen-containing molecules displaced with 2M ammonia solution. The pH of the eluents was measured to ensure sufficient resin and ammonia were used. Additional separations were carried out on the nitrogen-containing fractions using strongly basic anion exchange resin Dowex 1 in the hydroxide form, eluting with water, and then displacing retained acidic and neutral nitrogen-containing compounds with 2M HOAc.

### 4.2. Chromatography–Mass Spectrometry (GC-MS)

The dried samples were reacted with 30 µL of Pierce TriSil reagent in sealed GC vials and Whirlimixed; after heating at 50 °C for 20 min, the trimethylsilylated (TMS) samples were centrifuged and analyzed on a Perkin Elmer Clarus spectrometer using a high-polarity fused-silica column (Perkin Elmer Elite—5MS 30 m 0.25 mm ID 0.25 umdf). The carrier gas (helium) flow rate was 1 mL/min. Injections of 1ul were performed via an injector (200 °C) liner and the TMS derivatives were separated using a temperature program starting at 160 °C for 5 min, followed by a linear increase to 300 °C at a rate of 10 °C/min. Electron impact mass spectrometry of the column eluent was carried out with the quadrupole ion filter system run at 250 °C constantly during analysis. The detector range was set from 100 to 650 amu with a 3 min filament delay.

### 4.3. Nuclear Magnetic Resonance (NMR)

The 500 MHz NMR spectra were run after dissolving the samples in 550 µL of DMSO-d_6_ using an Avance spectrometer, Bruker UK Limited, Coventry, UK.

### 4.4. Glycosidase Inhibition Assay

All enzymes and para-nitrophenyl substrates were purchased from Sigma. Enzymes were assayed at 27 °C in 0.1 M citric acid/0.2 M disodium hydrogen phosphate buffers at the optimum pH for the enzyme. The incubation mixture consisted of 10 µL enzyme solution, 10 µL of 10 mg/mL aqueous solution of extract (in water), and 50 µL of the appropriate 5 mM para-nitrophenyl substrate made up in buffer at the optimum pH for the enzyme. The reactions were stopped by the addition of 70 µL 0.4 M glycine (pH 10.4) during the exponential phase of the reaction, which had been determined at the beginning using uninhibited assays in which water replaced the inhibitor. Final absorbances were read at 405 nm using a Versamax microplate reader (Molecular Devices). Assays were carried out in triplicate, and the values given are means of the three replicates per assay. The method is described by Watson and coauthors [41].

## 5. Conclusions

*Amorpha fruticosa* is a promising source of bioactive compounds for diabetes treatment. It contains stilbenes and these metabolites can be inhibitory to yeast alpha-glucosidase as well as porcine alpha-amylase and human pancreatic lipase. We here report on further analysis and activity assessments of *A. fruticosa* pods and leaves collected in Bulgaria, and for the first time have discovered a wider range of glycosidase inhibitions due to nitrogen-containing molecules. Pinitol and hydroxylated pipecolic acids are reported for the first time in the species as well as more complex iminosugar-like compounds that may all contribute to antidiabetic potential. Hydroxylated pipecolic acids are probable precursors of iminosugars and are common in legumes containing them. Considerable chemical variation was observed over the four pod collections.

Based on the results reported here, there should be further research to test the toxicity of water extracts at different concentrations and to define the best period for plant material collection for the optimal content of glycosidase inhibitors. If proven safe, the use of *A. fruticosa* pods may not only contribute to the treatment of diabetes, but could reduce the uncontrolled spread of the diaspores and invasive behavior of this plant. Its sustainable use can be established with beekeepers, who can benefit from its high pollen and nectar content, and after the blooming periods the pods can be harvested for antidiabetic applications. It would be worth analyzing the *Amorpha* honey for iminosugars and pipecolic acids related to glabrin, which has been reported to have antibacterial properties.

## Figures and Tables

**Table 1:** Extractable weight per gram. Legend: 1-*A. fruticosa* pods collected from the locations 1–4 and leaves from location 5.

	Pods				Leaves
Extractables	1	2	3	4	5
50% aq ethanol extractable	232 mg	231 mg	171 mg	161 mg	167 mg
Methanol extractable	146 mg	158 mg	121 mg	116 mg	
Total amino acids	2.5 mg	3 mg	2.4 mg	2 mg	6 mg
Pinitol	30 mg	23 mg	10 mg	5 mg	100 mg
Hydroxyhygrinic acid	0.23 mg	0.02 mg	0.009 mg	0.007 mg	1 mg
5-hydroxypipecolic acid	0.12 mg				1 mg
4,5-dihydroxy-*N*-methyl-pipecolic acid (tentative)	0.1 mg	trace	trace	0.008 mg	
Glutamic acid	0.22 mg	0.3 mg	0.5 mg	0.3 mg	0.3 mg
Proline	0.13 mg	0.17 mg	0.09 mg	0.26 mg	1.5 mg
Tyrosine	0.7 mg	0.34 mg	0.5 mg	0.3 mg	1.5 mg
2-aminoadenine-N1-oxide		0.8 mg	0.3 mg	0.14 mg	not evident
Myoinositol	3.5 mg	3.7 mg	3.2 mg	3.2 mg	

**Table 2:** Glycosidase inhibition of extracts and cation exchange resin-retained fractions of *A. fruticosa* pods (extract of 4) and leaves shown as % inhibition.

Type	Conc	α-D-Glucosidase	α-D-Glucosidase	β-D-Glucosidase	α-D-Galactosidase	β-D-Galactosidase	α-D-Mannosidase	N-Acetyl-β-D-Gluc	β-Glu-curonidase
		Yeast	Rat Intestine	Almond	Green Coffee Beans	Rat Intestine	Rat Intestine	Rat Intestine	Bovine Liver
pod methanol	10 mg/mL	96.6	2.4	67.4		16.2	−11.2	61.9	92
pod methanol	1 mg/mL	84.8						10.2	65.6
pod methanol	0.1 mg/mL	30.2						ND	13.2
pod 50% aq	10 mg/mL	96.2	6.6	77.1		18.4	−13.9	91.3	93.7
pod 50% aq	1 mg/mL	96.9						28.1	66.9
pod 50% aq	0.1 mg/mL	60.5						ND	12.3
pod cation-bound	10 mg/mL	92.3	4	50.6		3	−1.8	43.7	68.7
pod cation-bound	1 mg/mL	51.3						9.2	7.4
pod cation-bound	0.1 mg/mL	17.3						ND	4.2
pod bd Dowex 1	10 mg/mL	0.3	−0.2	19.5	−17	12.3		3.6	1
pod unbd Dowex 1	10 mg/mL	42.1	13.9	21.7	0.2	14.9		33.2	49.6
seedling cation-bound	10 mg/mL	41.7						27.2	11.4
leaf anion 5-unbound	10 mg/mL	10.9	54.2	9	7.9	−0.2		−3.3	−1.9
leaf anion-unbound end	10 mg/mL	8	36.2	10.1	−0.4	2.5		−3.6	1.3
leaf anion-bound	10 mg/mL	−4.9	0.7	9.1	−5.6	−6.1		5	−5.2

## Data Availability

Data are contained within the article and Supplementary Materials.

## Supplementary Materials

The following supporting information can be downloaded at https://www.mdpi.com/article/10.3390/plants14142205/s1.
