# Supplementary material for: Iminosugars of the Invasive Arboreal Amorpha fruticosa and Glycosidase Inhibition Potential
Source: Plants (Basel). 2025 Jul 16;14(14):2205. doi: 10.3390/plants14142205 (PMC12299671; doi:10.3390/plants14142205)
Supplement: Supplementary file 1 [file plants-14-02205-s001.zip › plants-3711933-supplementary.pdf]

**Supplementary Information**

# Iminosugars of the invasive arboreal *Amorpha fruticosa* and glycosidase inhibition potential

**Robert J. Nash [1], Barbara Bartholomew [1], Yana B. Penkova [1], and Ekaterina Kozuharova[2,*]**

A compound identified as a 5-hydroxypipecolic acid on the basis of GCMS was seen in *A. fruticosa* leaves and seeds. It was retained by an anion exchange resin confirming the presence of a carboxyl group. The compound gave a good match in the NIST library spectrum and with the 2*S*,5*R*-hydroxypipecolic acid reference spectrum of PhytoQuest compound 900439 (from *Calliandra cumingii* seeds) run on the same instrument. The molecular weight of the presumed pipecolic acid is 145 amu which with 3 trimethylsilyl-groups to give fragments 361 – 15 amu (methyl) to give 346 amu and then -COOtms gives 244 amu.

**Mass spectrum of 5-hydroxypipecolic acid (tms) seen in *A. fruticosa* seeds**

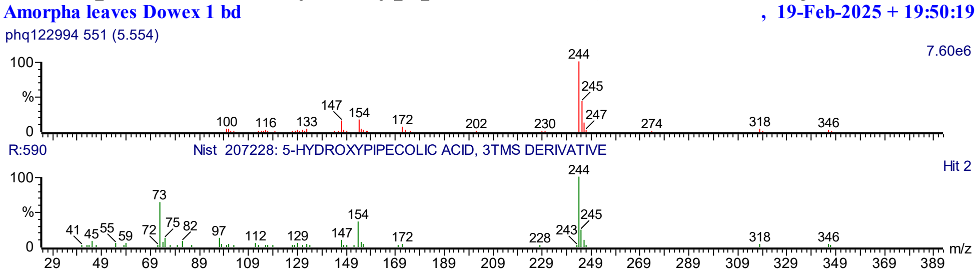


**Mass spectrum of the PhytoQuest reference compound (900439)**

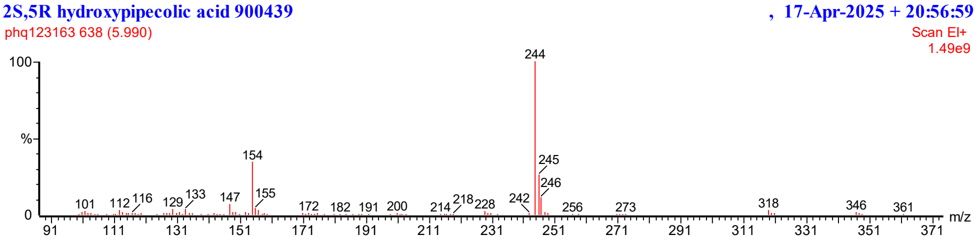


The *A. fruticosa* hydroxy-pipecolic acid has a retention time of 5.55 minutes which is shorter than the reference compound of 2*S*, 5*R*-hydroxypipecolic acid (PhytoQuest 900439) and so tentatively could be another epimer and tentatively 2*S*, 5*S*-hydroxypipecolic acid.

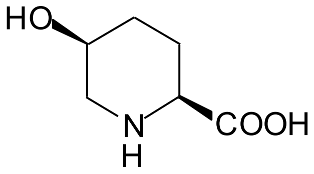


2*S*,5*S*-pipecolic acid

A higher molecular weight pipecolic acid was also seen with a longer retention time and tentatively identified as a dihydroxy-pipecolic acid with a distinctive mass spectrum but was not similar to PhytoQuest reference compound 900438 (2*S*, 4*S*, 5*R*)-dihydroxypipecolic acid or compound (2*S*,4*S*,5*S*)-dihydroxypipecolic acid (PhytoQuest synthetic compound 900287).

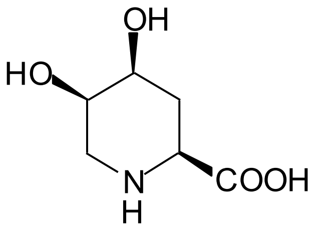


PhytoQuest 900438
*Calliandra cumingii*

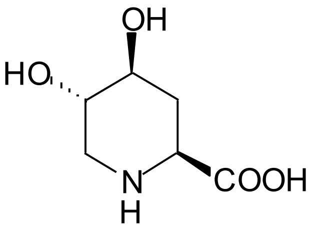


9000287
synthetic

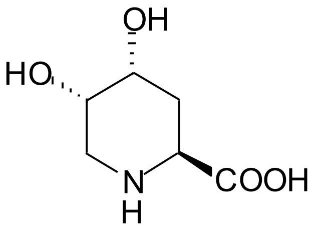


900454
*Inga squamigera*

**Mass spectrum of 2*S*, 4*S*, 5*R*-dihydroxypipecolic acid (PhytoQuest reference compound 900438)**

2S,4S, 5R dihydroxypipecolic acid 900438 , 17-Apr-2025 + 21:29:30
phq123164 1157 (8.586) Scan EI+ 2.36e8

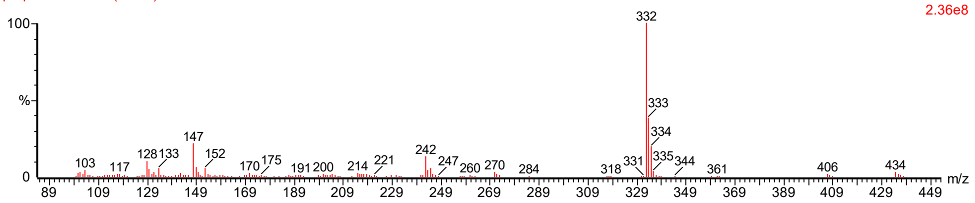


**Mass spectrum of 2*S*,4*S*,5*S*-dihydroxypipecolic acid (tms) (PhytoQuest reference compound 900287)**

**HS0177/108/1** **, 10-Jul-2008 + 14:11:11**
SUM237105 1164 (8.989) Scan EI+ 6.17e7

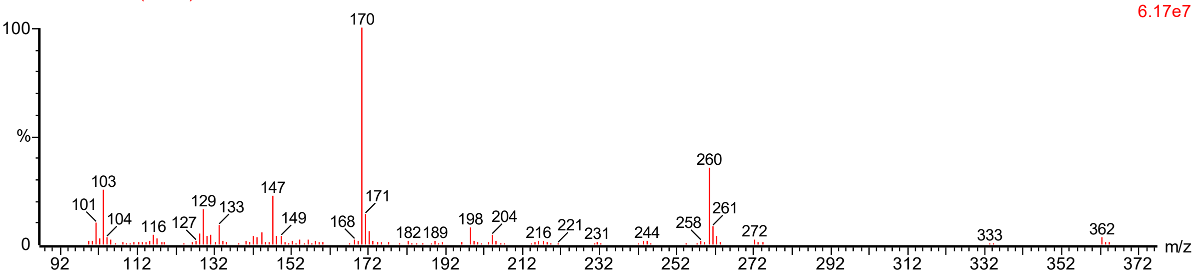


**Mass spectrum of 2R,4S,5R-dihydroxypipecolic acid (tms) (PhytoQuest reference compound 900454)**

**JH0809/52/7** **, 28-Mar-2011 + 19:39:45**
PHQ023415 1023 (8.260) Scan EI+ 1.62e6

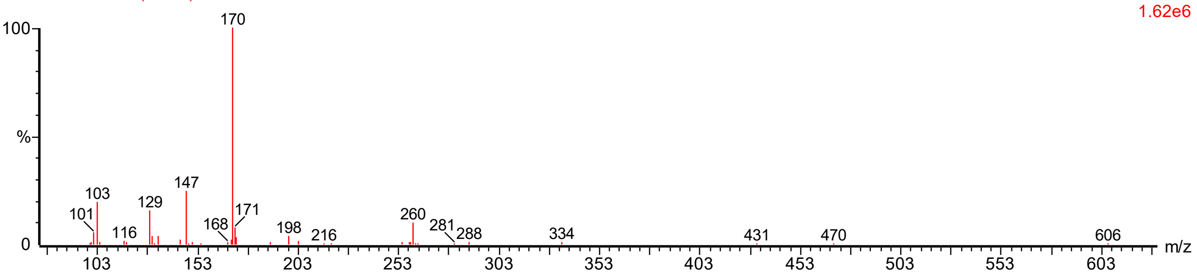

**Mass spectrum of the presumed dihydroxypipecolic acid from *A. fruticosa* seeds with retention time of 8.56 minutes suggested it had a methyl-substituent.**

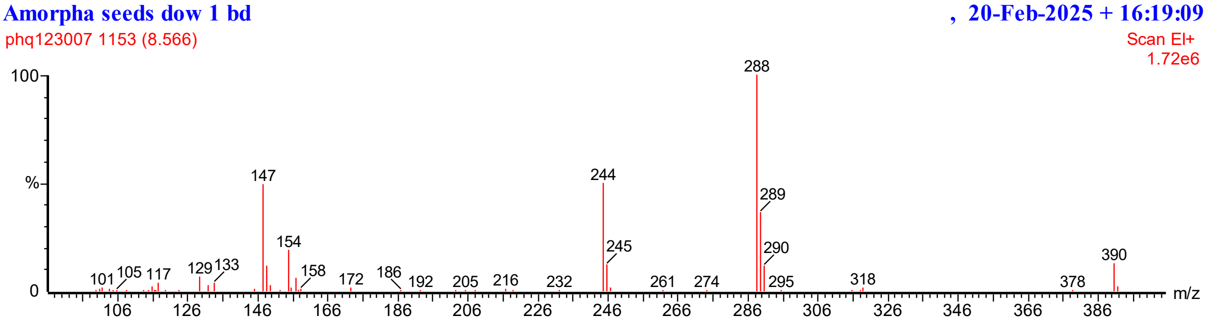


The structure is tentatively the *N*-methyl-2*S*, 4,5-dihydroxypipecolic acid. The position of the extra hydroxyl and stereochemistry of the hydroxyls cannot be confirmed without isolation and NMR analysis. This molecule gives a molecular ion at 390 corresponding to 3 trimethylsilyl-groups and a methyl which is most likely on the nitrogen (apparent loss of 14 from 288 to 274 amu?) but could be on one of the hydroxyls.

Another tentative minor iminosugar acid was observed at 7.79 minutes in *A. fruticosa* seeds. It gave a similar mass spectrum to a dihydroxy-2-methyl-2-carboxy-proline (PhytoQuest 900407) but that is synthetic and unlikely to be the same compound in seeds. The mass spectrum also has similarities to that of the pipecolic acid 900287 shown above.

**Mass spectrum of unknown minor iminosugar acid (tms) retained by anion exchange resin from seeds**

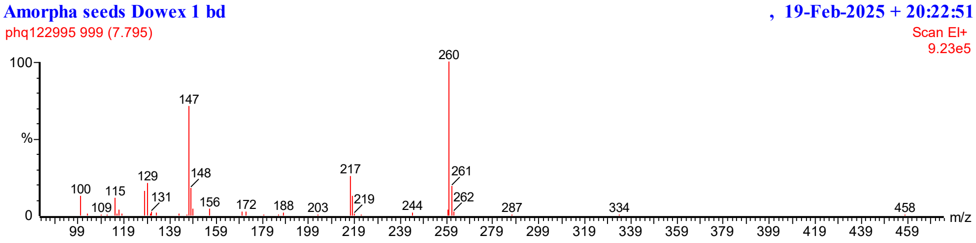


**PhytoQuest library match and mass spectrum of unknown minor iminosugar acid**

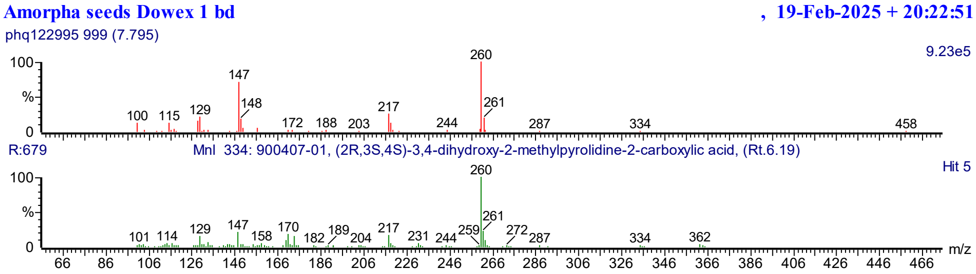


Glabrin is a *N*-methyl-dihydroxypipecolic acid reported from *Pongamia pinnata* and claimed to be anti-bacterial (Bhatt, G., Singh, A., Panda, A.N. *et al.* Glabrin from *Pongamia pinnata*: Structural Insights and Antibacterial Potential. *Natl. Acad. Sci.*

*Lett.* **48**, 27–31 (2025). A possible isomer of glabrin was seen in *A. fruticosa* seeds on the basis of a similar mass spectrum but different retention time.

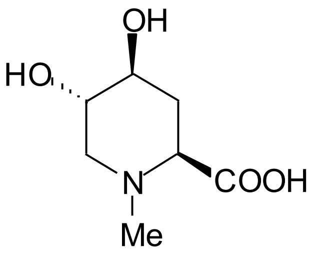


glabrin (PhytoQuest compound 900429)

## Mass spectrum of tentative glabrin isomer in *A. fruticosa* seeds

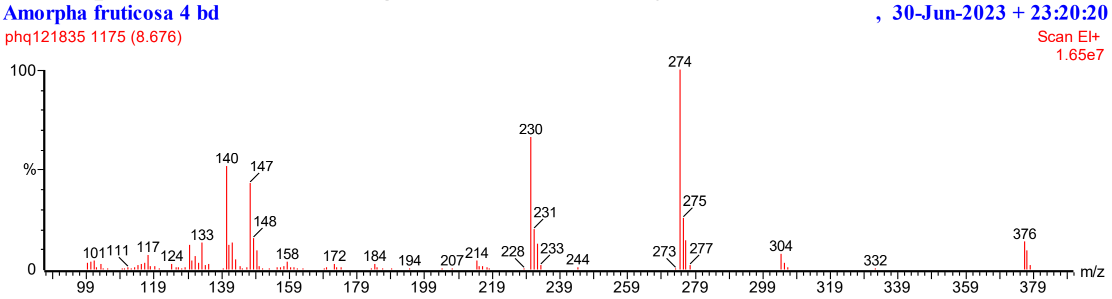


## Reference mass spectrum of reference glabrin (tms) (900429)

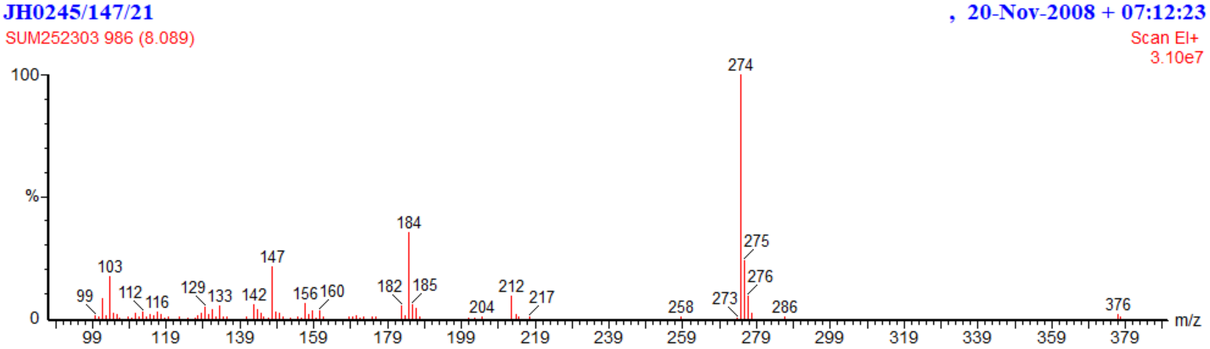


## Mass spectrum (tms) of small iminosugar imino acid observed in *A. fruticosa* seeds tentatively identified as 4-hydroxyhygrinic acid

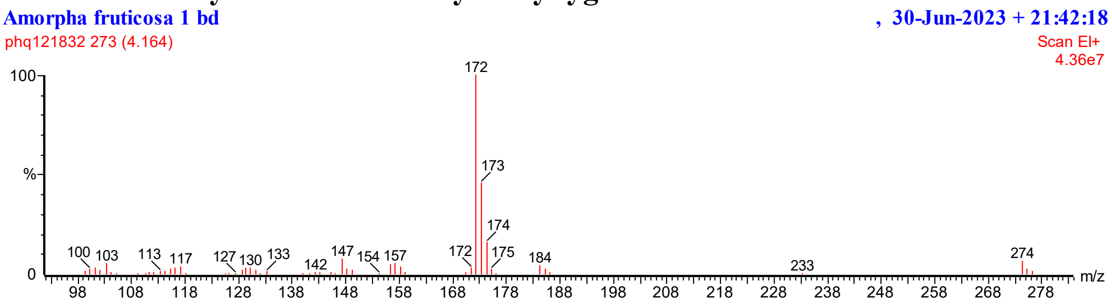

## Mass spectrum of 4-hydroxyhygrinic acid (tms) (PhytoQuest compound 900096)

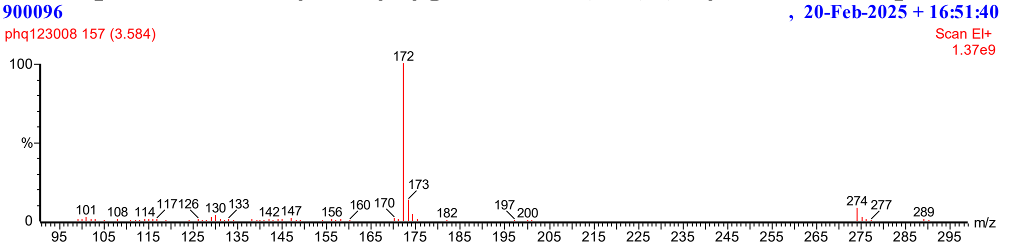


The adenine derivative 2-aminoadenine-N1-oxide was tentatively identified in *A. fruticosa* seeds by the distinctive mass spectrum and by comparison with a reference spectrum of PhytoQuest compound 900092. This compound is also reported from *Trifolium pratense*.

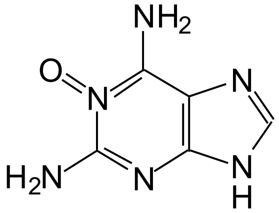


## Mass spectrum of 2-aminoadenine-N1-oxide (tms) seen in *A. fruticosa* seeds

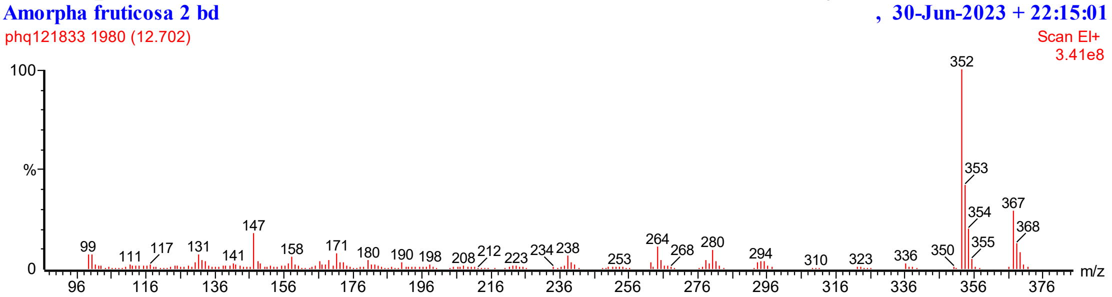


## Reference spectrum of 2-aminoadenine-N1-oxide (tms) (PhytoQuest 900092)

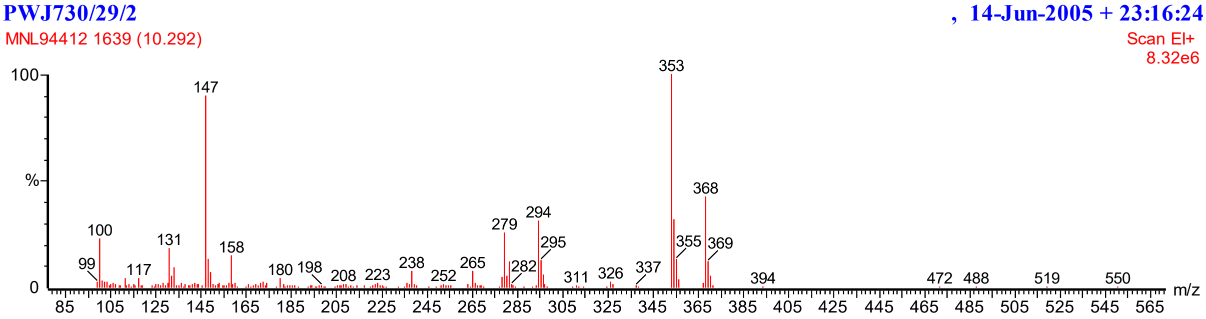


## Mass spectrum of another probable novel iminosugar acid (tms) seen in *A. fruticosa* seeds and possibly derived from proline

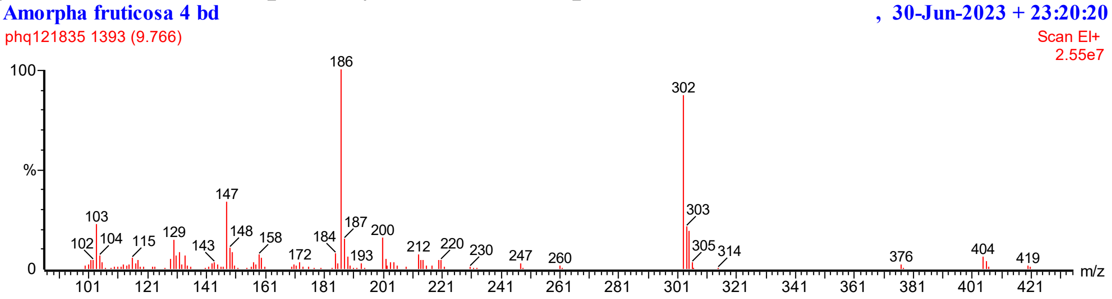

## Mass spectrum of proline (tms) seen in *A. fruticosa* seeds

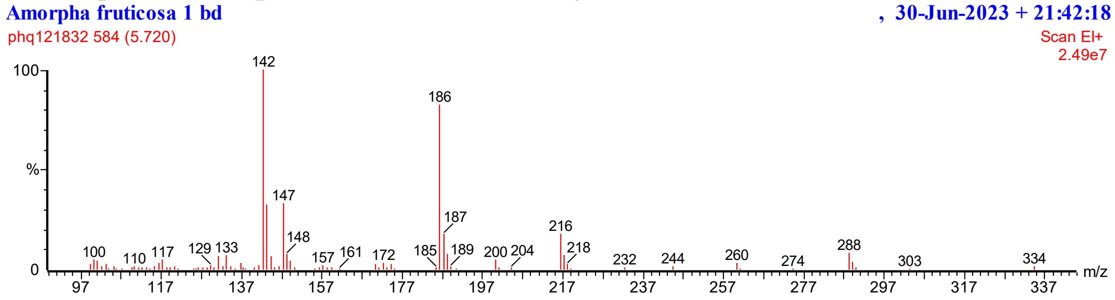


## Mass spectrum of pinitol (tms) seen in *A. fruticosa* seeds and leaves

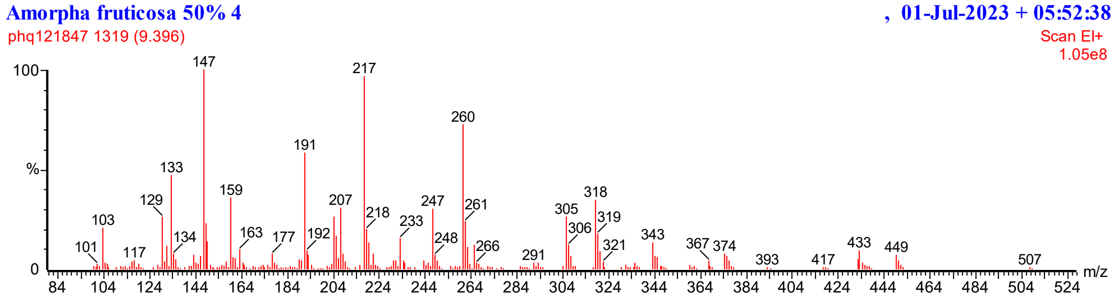


## Mass spectrum of gallic acid (tms) seen in seeds of *A. fruticosa* at low concentration

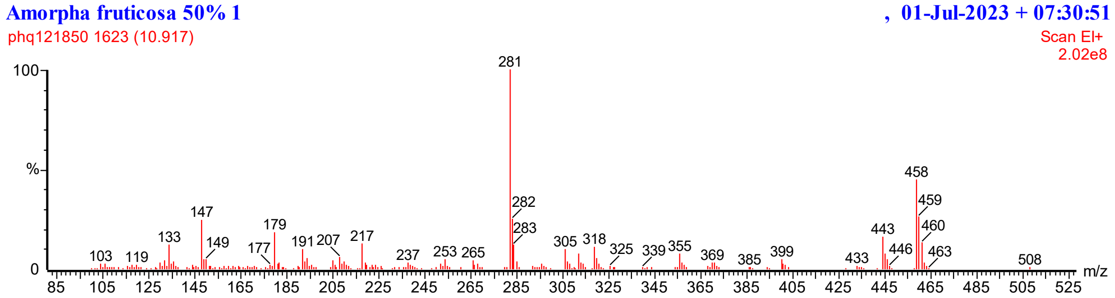


## Table showing % inhibition of glycosidases by PhytoQuest reference iminosugar acid compounds tested at 0.4mM

| Phyto Quest code | α-D-glucosidase | α-D-glucosidase | α-D-glucosidase | β-D-glucosidase | α-D-galactosidase | β-D-galactosidase | α-L-fucosidase | α-D-mannosidase | β-D-mannosidase | Naringinase | N-acetyl-β-D-gluc | N-acetyl-β-D-gluc | β-glucuronidase |
|---|---|---|---|---|---|---|---|---|---|---|---|---|---|
| | Yeast | *Bacillus* | Rice | Almond | Green coffee beans | Bovine liver | Bovine | Jack bean | *C. fimi* | *P. decumbens* | Bovine kidney | Jack bean | Bovine liver |
| 900092 | 0 | -4 | 9 | 0.3 | -4 | 45 | 4 | 5.7 | 3.4 | 9.3 | -4.7 | -3.9 | ND |
| 900096 | -1.2 | 7.4 | 4 | 7.1 | 0 | -5 | 14.5 | 6 | -4 | 3 | -5 | 1.6 | -3.7 |
| 900287 | 4.2 | 12.9 | | 1.3 | -1.8 | 1.7 | | -20.1 | -6.7 | | 3.5 | -7.4 | 14 |
| 900407 | 1.4 | -0.2 | | 0.6 | -9 | -5.2 | | -6.4 | -6 | | 7.6 | -7.9 | 0.6 |
| 900429 | -2.7 | -4.2 | | 4.1 | 0.2 | 2.3 | | -14.3 | -2.4 | | -0.9 | -2.8 | 5.6 |
| 900438 | -3.4 | -4.5 | 0 | -4.1 | -6.6 | -3.7 | | 4.8 | -6.5 | -6 | 0.5 | -0.3 | -4.2 |

It can be seen that pipecolic acids and the adenine derivative do not give strong inhibition of the glycosidases used in the above screen although 900092 does give selective inhibition of the β-galactosidase and dihydroxypipecolic acid 900287 gives some inhibition of a Bacillus α-glucosidase and also inhibits a bovine β-glucuronidase. In the results a negative value suggests a promotion/stabilisation but if less than -10 it is probably not a notable effect.
